# Past climate variations recorded in needle-like aragonites correlate with organic carbon burial efficiency as revealed by lake sediments in Croatia

**DOI:** 10.1038/s41598-021-87166-2

**Published:** 2021-04-07

**Authors:** Ivan Razum, Petra Bajo, Dea Brunović, Nikolina Ilijanić, Ozren Hasan, Ursula Röhl, Martina Šparica Miko, Slobodan Miko

**Affiliations:** 1 Croatian Natural History Museum, Demetrova 1, 10000 Zagreb, Croatia; 2 Croatian Geological Survey, Sachsova 2, 10000 Zagreb, Croatia; 3 MARUM - Center for Marine Environmental Sciences, University of Bremen, Leobener Strasse 8, 28359 Bremen, Germany

**Keywords:** Biogeochemistry, Climate sciences, Limnology

## Abstract

The drivers of organic carbon (OC) burial efficiency are still poorly understood despite their key role in reliable projections of future climate trends. Here, we provide insights on this issue by presenting a paleoclimate time series of sediments, including the OC contents, from Lake Veliko jezero, Croatia. The Sr/Ca ratios of the bulk sediment are mainly derived from the strontium (Sr) and calcium (Ca) concentrations of needle-like aragonite in Core M1-A and used as paleotemperature and paleohydrology indicators. Four major and six minor cold and dry events were detected in the interval from 8.3 to 2.6 calibrated kilo anno before present (cal ka BP). The combined assessment of Sr/Ca ratios, OC content, carbon/nitrogen (C/N) ratios, stable carbon isotope (δ^13^C) ratios, and modeled geochemical proxies for paleoredox conditions and aeolian input revealed that cold and dry climate states promoted anoxic conditions in the lake, thereby enhancing organic matter preservation and increasing the OC burial efficiency. Our study shows that the projected future increase in temperature might play an important role in the OC burial efficiency of meromictic lakes.

## Introduction

Lakes have disproportionally large annual amounts of buried organic carbon (OC) compared to oceans^1^ and are of great importance for the global carbon budget and cycle; thus, they might also have a vast impact on climate change in the future. However, the influence of climate on OC burial efficiency is still not precisely understood. The influence of temperature on the OC burial efficiency has been studied extensively, mainly for lakes at higher northern latitudes^2–6^. Although a large number of studies have focused on this topic, a consensus has not been reached on the possible influence of temperature on the OC burial efficiency. Moreover, the main driving mechanisms that are directly responsible for the observed changes in OC content of lake sediments have not been identified. Studies have inferred that higher temperatures enhance OC burial mainly as a consequence of denser vegetation cover^3,7–10^, with an increase in temperature positively correlated with OC mineralization^2,9,11^. In more recent studies, anthropogenic influences have been reported, primarily through the role of reactive nitrogen and phosphorus on OC burial efficiency^4,6,12^, and the temperature effect has been negated. Furthermore, oxygen exposure time and redox conditions in the water may also play a prominent role in the OC burial efficiency^5,9,13,14^.

**Figure 1:**
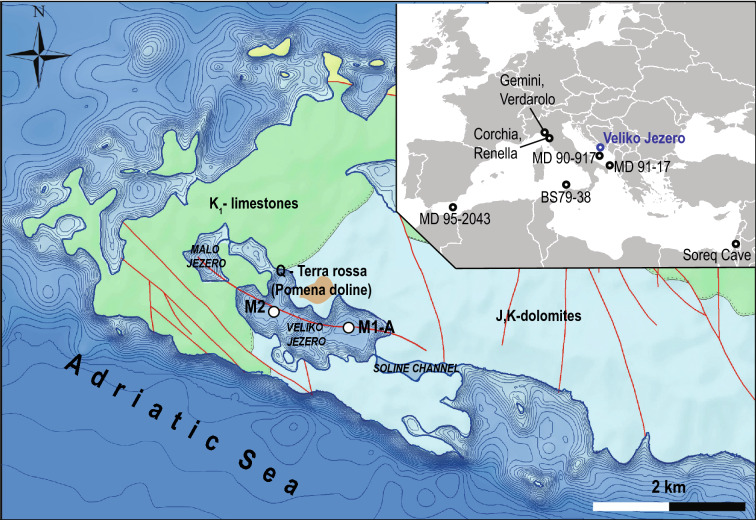
Location of Lake Veliko jezero and Mediterranean climate records (as discussed in the text) in the upper right corner with the locations of Cores M1-A and M2 and the stratigraphical and lithological surroundings of Lake Veliko jezero. Map was created using the ESRI ArcInfo 10.2.1 GIS software (https://www.esri.com/).

**Figure 2:**
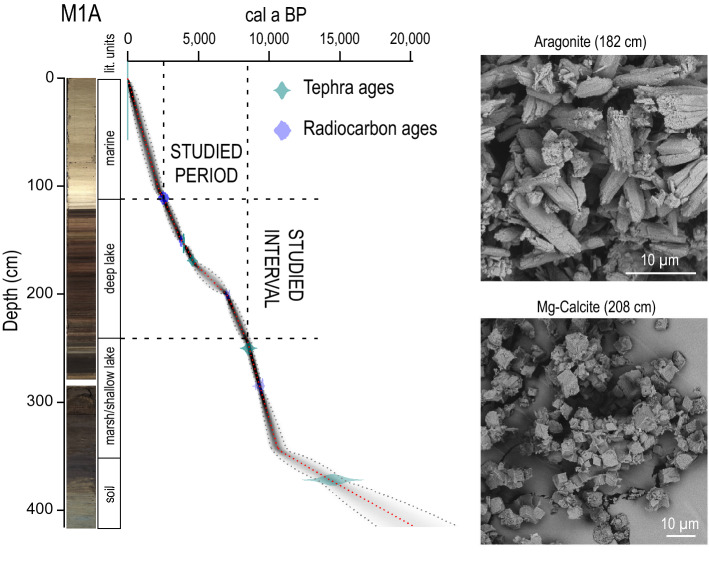
Age-depth model for Core M1-A from Lake Veliko jezero, with core images and lithological units shown next to the depth scale. SEM images of inorganic aragonite and Mg-calcite are shown on the right-hand side.

The majority of previous studies on OC burial efficiency have focused on recent and subrecent lake sediments, where climate effects can easily be concealed by anthropogenic influences. Additionally, in latitudinal studies, the temperature effect can be masked by other variables, such as changes in vegetation cover and/or precipitation rate. To infer the climate influence on OC burial efficiency, we studied sediments of Lake Veliko jezero, Croatia (Fig. 1), for the period 8.3 to 2.6 cal ka BP. In detail, we interpreted the OC content in the context of high-resolution relative paleoclimate (log ratio of Sr/Ca) and paleoredox proxies (log ratio of Mo/detrital elements) and as an indicator for past aeolian activity (log ratio of Zr over Al). Our approach for correlating paleoclimate and paleoredox proxies was performed by considering the properties of compositional data^15^, which enabled more precise interpretations. The Sr/Ca ratios of the bulk sediment are mainly derived from Sr and Ca concentrations of needle-like aragonite in Core M1-A and used as paleoclimate, temperature proxies, with higher ratios indicating cooler conditions. The Sr/Ca ratio of aragonite is typically used as a proxy for sea surface temperature (SST) variability^16^ because incorporation of Sr into aragonite coral skeletons as well as in inorganic aragonite is temperature dependent, i.e., an increase in temperature lowers the Sr/Ca ratio in inorganic aragonite^17,18^ and coral skeletons^19^. However, this simplistic interpretation has been challenged because it has been proven that incorporation of Sr into aragonite corral skeletons is also affected by vital effects^20,21^, variability of the Sr/Ca ratio in the oceans^22,23^ and unwanted effects caused by algal symbionts^24^. Compared with numerous paleoclimate studies of biogenic aragonite, the paleoclimate potential of inorganic aragonite in marine/lake sediments needs to be explored, which is mainly because sedimentary environments in which inorganic aragonite precipitates are rare, and studies show that the Sr/Ca ratio of inorganic aragonite is much less sensitive to temperature changes than the Sr/Ca ratio of coral aragonite skeletons^21^. Despite this obstacle, the main advantage of inorganic aragonite is the lack of vital effects. We validated the reliability of this novel proxy by also acquiring the same kind of data for Core M-2 that was taken from a different basin of the same lake. An age-depth model of Core M1-A (Fig. 2) was previously described^25^. The significance of the Sr/Ca ratio as a paleotemperature proxy was also confirmed by correlation with other previously published studies, including the main Holocene climatic events from the wider Mediterranean region^26–31^.

## Results and discussion

### Study site and core description

Lake Veliko jezero, with a surface area of 1.44 km^2^, consists of three basins; core M1-A is derived from the deepest basin, with a maximum depth of 46 m (Fig. 1). Core M2 was retrieved from the second largest basin from a depth of 40 m. The lake is located on the island of Mljet (42°44´ N; 17°31´E), which is a part of the Adriatic carbonate platform^32^ (AdcP). Late Jurassic and Early Cretaceous dolomites are exposed around the lake. The lake is part of the karst depression system, which is now a submerged sinkhole due to Holocene sea level rise. At the early Holocene, 10.8 cal ka BP^25^, because of the sea level rise and the presence of permeable karst, a wetland formed that transitioned into a brackish lake and finally ended as a marine lake because of sea flooding through the Soline channel (2.5 m deep) 2.3 cal ka BP^25^. The lake was perennial, with an almost constant salinity of ~25‰ during the studied interval^33^ (8.3–2.6 cal ka BP). The only connection with the sea was through permeable karst, while the only source of freshwater was local precipitation.

Core M1-A recovered from Lake Veliko jezero is 417 cm long, measured from the lake bottom, and four lithological units are distinguished (Fig. 2). The top unit (0–108 cm) is made up of gray homogenous marine sediment. The underlying unit (108–241 cm) consists of deep lake sediment characterized by alternations of white and dark laminas mainly composed of aragonite and organic matter. The top part of that unit consists of a few centimeters of oxidized lake sediment. The following unit from 241 to 343 cm is marsh to shallow lake sediment. Finally, the bottom unit (343–417 cm) is terra rossa-type soil interbedded with thick tephra. Core M2 resembles Core M1-A with the difference that unit boundaries are at different depths. Therefore, the studied interval spans from 127 to 266 cm in this core.

### High-resolution XRF scanning and age-depth model

**Figure 3:**
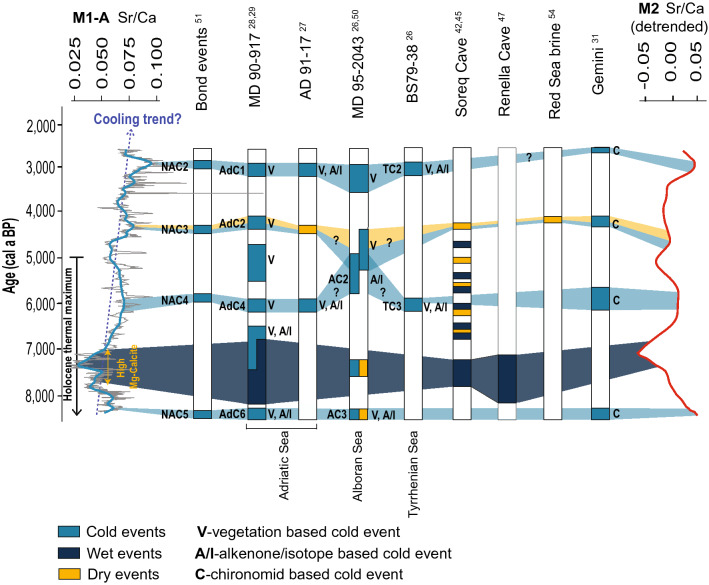
Sr/Ca records for Cores M1 and M2 as indicators of relative paleotemperature and paleohydrology compared to paleotemperature and paleohydrological proxies from other records from the wider Mediterranean region. The Mg-calcite-rich interval is underlain by a dark blue area in the Sr/Ca record. Otherwise, needle-like aragonite is the dominant phase. NAC, AdC, AC, and TC are abbreviations for cold events based on the original publications.

The Sr/Ca records were obtained by high-resolution XRF core scanning at split core surfaces at 1 cm and 2 mm resolutions. We compared the Sr/Ca data and ratios between the two investigated cores (M1-A and M2) to confirm the robustness of our record and exclude any potential analytical artifacts. M1-A core chronology is based on four tephra layers and four C-14 dates^25^. Briefly, the studied interval chronology is very well constrained since in ~1.25 m, there are three tephra layers, which serve as depth-age model anchor points: two charcoal C-14 dates free of reservoir effects and one marine shell C-14 date with known reservoir effects (Fig. 2). The geochronological age of Core M2 is based on one plant remaining sample, which was dated by radiocarbon methodology. Three additional data points were included in the age-depth model of this core. Two of these ages are based on visible tephra layers correlated to the known volcanic eruptions previously recognized in Core M1-A^25^. The third date corresponds to a time of marine intrusion into the lake, which is marked by a sharp boundary between the top and underlying lithological units (Fig. 2) in both cores. It was radiometrically dated in Core M1-A^25^. Individual radiocarbon dates were calibrated using the R package rbacon^34^ (the age-depth model for core M2 is presented in the Supplementary file, Figure S4). Despite the lower resolution of the Core M2 record, it is evident that major peaks and troughs in Sr/Ca are well presented in both datasets (Fig. 3).

### Sr/Ca ratio as paleoclimate proxy

**Figure 4:**
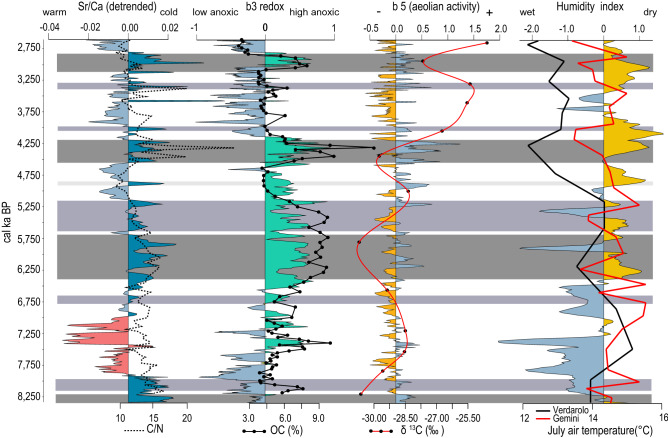
Sr/Ca (3-point average curve) balances b3 (redox) and b5 (aeolian input) derived from the XRF data and its connection with organic matter data (C/N, OC and δ^13^C). Gray bands indicate the main cold events, and purple bands are minor cold events recognized in M1-A. The correlation of those events with temperature changes in Lakes Gemini and Verdarolo and the humidity index from Corchia are visible on the right side of the figure.

Our working hypothesis was that the Sr/Ca ratio in bulk sediment almost exclusively reflects the Sr/Ca ratio of needle-like aragonite in the sediment (Fig. 2). The temperature and Mg/Ca ratio are the two most important variables for the precipitation of inorganic aragonite^35^. The main source of Mg for the aragonites from the Veliko jezero lake could be seawater, and the second source could be from the surrounding carbonate rocks, which are mainly dolomites. Although the Mg/Ca ratio does not affect the crystallization of aragonites directly, it prevents the formation of calcite and the transformation of aragonite into calcite^36–38^. Additionally, the Sr/Ca ratio in aragonite is not affected by variations in salinity, sulfate or carbon dioxide content^37,39,40^. Needle-like aragonite currently precipitates during late spring and summer in the adjacent Lake Malo jezero^41^. Since the Sr/Ca ratio of needle-like aragonite is largely temperature dependent^17,18^, we assume that the Sr/Ca ratio of the studied sediment could be used as a relative paleotemperature proxy. Mineralogical analysis performed with X-ray diffraction (XRD) and scanning electron microscopy coupled with energy dispersive spectroscopy (SEM–EDS) proved that inorganic needle-like aragonite is the main mineral and nearly the only carbonate phase in our investigated samples (Fig. 2). The only exception is an interval from 201 to 214 cm, where inorganic rhombohedral Mg-calcite is the main carbonate phase, thus confirming previously published results^33^. According to the age model of Core M1-A, the occurrence of Mg-calcite coincides with known time intervals of wet climate during which lake levels were high and generally correspond to pluvial periods observed in the wider Mediterranean region (Figs. 3 and 4, from ca. 7.6 to 7 ka BP)^28–30,42–47^. We propose that during this period, increased freshwater input lowered the Mg/Ca ratio in the lake water, leading to Mg-calcite precipitation and hindering the precipitation of aragonite. This consequently led to a Sr/Ca decrease in our records because Mg-calcite incorporates Sr in the crystal lattice less effectively than aragonite^48^; thus, the Sr/Ca ratio of bulk sediment cannot be interpreted as a relative paleotemperature proxy in this interval. However, the predominance of Mg-calcite over aragonite points to wetter climate conditions, which are also observed in the wider region during this time period^28–30,42–47^.

To utilize the Sr/Ca ratio as a relative paleotemperature proxy, we have proven that detrital Sr and Ca inputs are negligible. XRD analyses revealed aragonite as the main mineral, with minor quartz content limited only to the oldest portion of the studied period. Furthermore, large variances of the centered log ratio transformed variables of Sr and Ca relative to detrital elements, such as titanium (Ti) and aluminum (Al) (Supplementary file, Figure S2) combined with small logratio varaince of Sr and Ca relative to the inorganic carbon (INC) (Supplementary file, Figure S2) additionally confirmed that Sr and Ca represent carbonate components and are not of detrital origin.

Finally, the Sr budget of the Lake Veliko jezero water and consequently the Sr/Ca ratio of the bulk sediment can also be influenced by hydrological variability. Changes in hydrological regime would theoretically affect the relative marine influence at this location because of a limited connection of the lake to the Adriatic Sea through permeable karst: ocean water is characterized by higher Sr concentrations than freshwater^49^. Two possible scenarios emerge if hydrologically induced Sr availability was the limiting factor for the Sr/Ca ratio of the bulk sediment.

First, during cold periods, Sr/Ca in the lake water would be lower because of the reduced evaporation rate, i.e., decreased marine influence, which would finally result in a relative decrease in the Sr concentration of the lake water and consequently the Sr/Ca of the lake sediments. The opposite would be the case for warmer climate conditions. This scenario, however, can be discarded based upon the good correlation of the maxima in our Sr/Ca record with cold events that were previously observed in multiple paleoclimate archives in the Mediterranean region (Fig. 3). In Fig. 3, the locally estimated scatterplot smoothed (LOESS) Sr/Ca curve, with a smoothing factor of 0.09, displays four distinct peaks centered at 8.3, 6.0, 4.25 and 2.9 cal ka BP. The first Sr/Ca maxima in our record, which are centered at 8.3 ka, are coeval with cold events described in the pollen record from the Adriatic Sea^28^ and a decrease in the sea surface temperature^29^ recognized in the same core. In the Alboran Sea, a drop in the temperature during that time period was recorded as well^26,50^, while a minor drop in the temperature was also observed in Gemini Lake^31^. These events correlate with the North Atlantic cold event (NAC5)^51^. Following the pluvial period (7.6 to 7 cal ka BP) characterized by Mg-calcite deposition instead of aragonite, another maximum in the Sr/Ca record from the M1-A core, which is centered at approximately 6 cal ka BP, can be correlated with cold spells recognized in the Adriatic^27,28^ and Tyrrhenian and less clearly in the Alboran Sea^26^ as well as the NAC4 event in the North Atlantic^51^. Temperature reconstructions based on chironomid communities from Gemini and Verdarolo lakes also indicate cold conditions at approximately 6 cal ka BP^31^. A Sr/Ca maximum at approximately 4.25 cal ka BP is correlated with a decrease in the temperature in the Adriatic Sea and Italian lakes^28,31^ and in the north^51^. Finally, an increase in the Sr/Ca centered at approximately 2.9 cal ka BP correlates well with the cold spells already recognized in the Adriatic, Alboran and Tyrrhenian seas^26–28,50^ as well as in the North Atlantic^51^. A less pronounced temperature decrease during this time interval was observed in Lake Gemini, Italy^31^.

The second scenario implies that the environment during cold events was also dry, while during warmer events, it was wetter, which would lead to a decreased relative marine influence and the dilution of the Sr budget in the lake water. Such changes would finally result in lower Sr/Ca during warm periods and higher values during cold periods. To further investigate the role of wet versus dry conditions as potential drivers of Sr/Ca variability, we studied additional chemical elements acquired by XRF scanning, such as Br, Rb, Si, K, Na, Al, Fe, Mn, Zr, Ti and Mo, which were primarily validated by wet-chemical analysis on discrete samples, e.g., through inductively coupled plasma mass spectrometry (ICP-MS). We used all of these elements to model proxies called balances; for paleotemperature, aeolian input and paleoredox conditions via orthonormal log ratio transformation (OLR)^52^, which enabled firm statistical parameters to be established for reliable interpretations of the involved processes^53^. The methodology is fully explained in a previous study^53^ and described briefly in the Methods section. Ultimately, five balances were modeled, each of which is a proxy for a certain process: b3 and b4 are proxies for paleoredox conditions, b5 is a proxy for aeolian activity, and b2 is a proxy for paleoclimate Sr/Ca (rationale for the balance construction is shown in the Supplementary file, text S1, and sign matrix table used for OLR transformation is shown in the Supplementary file, table S1).

The data analyses show that an increase in Sr/Ca correlates with increases in anoxic conditions (*r*_*(b2–b3)*_ = 0.55, *p*_*(0.05)*_ = 0.00001) and aeolian activity (*r*_*(b2–b5)*_ = 0.56, *p*_*(0.05)*_ = 0.00001) (Supplementary file, table S2) (Fig. 4). A comparison between b2 and Sr/Ca is presented in the Supplementary file, figure S3). Furthermore, a speleothem-based paleohydrological reconstruction from Corchia cave in Italy^30^ implies that during the time corresponding to the Sr/Ca maxima in our record, climate conditions were generally drier (Fig. 4). However, it is also evident that the Sr/Ca ratios in our record do not exhibit exceptionally high values during the most widespread dry event in the Mediterranean and wider region at 4.2 ka^27,45,54–57^. This finding implies that hydrological conditions probably played less of a role in driving Sr/Ca ratios than the temperature effect. Following all lines of evidence, we propose that the Sr/Ca ratio of Lake Veliko jezero bulk sediment represents the Sr/Ca ratio of inorganic needle-like aragonite, which mainly reflects relative paleotemperature changes, while hydrological variability likely plays a secondary role.

One of the main advantages of XRF core scanning in addition to being nondestructive is the high resolution. If Sr/Ca ratios are a temperature indicator, then they may also be applicable for short events; for example, we observe several relatively brief cold events at 8.0, 6.6, 5.4, 4.8, 4.0 and 3.3 cal ka BP. From these, the events at 8.0, 6.6, 5.4 and 4.0 cal ka BP were also recorded in Lake Gemini while the event at 3.3 ka BP was recognized in Lake Verdarolo^31^. Most of these events are characterized by an increase in anoxic conditions and aeolian input, indicating not only cooler but also drier climate conditions at our sites (Fig. 4). These events can be detected due to the combined effects of the limited lake size and detrital influence on the studied lake(s) and the high resolution of the data. The small size of this lake (surface area of 1.44 km^2^) has very limited detrital influence, i.e., small effects of internal and landscape filters^58^ result in increased sensitivity in recording smaller-scale climate events.

### Disentangling the drivers of OC burial efficiency

To decipher the potential drivers of OC burial efficiency, we analyzed the OC, inorganic carbon (INC) and organic nitrogen (N) and explored their relationship with enhanced anoxic episodes, cold spells and aeolian input. Additionally, to better characterize the provenance of organic matter (OM), we analyzed δ^13^C throughout the same interval.

Higher C/N ratios (> 10) indicate potential mixing of land-derived and autochthonous organic matter^59,60^, while more negative δ^13^C values could also be related to the increase in the land-derived component of organic matter^61^. Our results demonstrate a slight long-term decreasing trend in the C/N record that coincides with a much stronger increasing trend of δ^13^C values. This anticorrelated pattern of the two proxies indicates that land-derived organic matter is partly decreasing, which is consistent with the overall detrital influence during our studied interval (Fig. 4). Additional evidence comes from the occurrence of quartz, which is only found at the base of the studied interval. A combination of two factors is most likely responsible for the described trends. First, lake deepening caused by the Holocene sea level rise moved the shoreline away from the core site. More specifically, the distance between Core M1-A and the Pomena doline, which is a small terra rossa soil patch adjacent to Lake Veliko jezero (Pomena field, Fig. 1), increased. Second, due to the Holocene sea level rise, a gradual increase in marine influence through permeable karst occurred. Sea level rise changed the lake biota^33^ and consequently the organic carbon content and composition.

During the studied interval, only two different pollen zones occurred, one with *Juniperus* and *Phillyrea* ca. 8 to 6.5 ka BP and another with *Quercus ilex* from 6.5 ka BP to the present^62^*.* This finding implies minor changes in terrestrial vegetation with negligible impacts on carbon content, composition and variability.

The correlation of the OC content with the Sr/Ca record reflecting temperature variations on the millennial time scale suggests that the OC content increased during cold exposure (Fig. 4). The observed OC increase during cold events is in line with a previous study^2^, where a temperature decrease leads to low mineralization of OC. However, the temperature effect through OC mineralization was not a substantial factor for OC preservation in the Lake Veliko jezero sediments. If temperature is a driver of OC preservation on millennial time scales, then cold climate conditions would decrease the degradation rate of algal (autochthonous) organic matter^63^, which would result in lower C/N values, higher OC and more positive δ^13^C values, which we did not observe in our data. Indeed, we observe slightly higher C/N and more negative δ^13^C values during cold spells, which would imply that land-sourced organic matter increased during cold spells as a result of enhanced aeolian input as confirmed by correlation analyses *r(b2-b5)* (Fig. 4). However, we argue that an increase in land-sourced organic matter is not the main mechanism underlying the overall OC increase because a more than double increase in OC during cold spells would have caused a substantial increase in C/N, which is not observed in our record. An exception is the 4.2 event, when maximum C/N ratios occurred.

The OC amount and variability also correlate with the paleoredox proxy (Fig. 4), i.e., an increase in anoxic conditions corresponds to an increase in OC content. Based on this finding, we propose that an increase in anoxic conditions is the main factor that led to OC preservation at our site. A decrease in temperature and possibly drier conditions during cold events would shift the redox zone boundary and thermocline closer to the surface^64^, which would prevent mixing of the water throughout the water body, thereby causing the anoxic boundary to move upward and leaving the majority of the water column under anoxic conditions. This supposition is also confirmed by the high correlation between paleotemperature (b2) and paleoredox (b3) proxies. Although the depth of a thermocline depends on a number of factors, such as the lake size, dissolved organic content, temperature, wind activity, etc.^65^. We believe that the temperature was the key factor controlling the thermocline and redox zone boundary depth in Lake Veliko jezero. These findings are underpinned by a study of modern processes in Lake Veliko jezero lake, where the thermocline occurs only during summer months^66^. Finally, a shallow thermocline/redox zone boundary would cause most of the organic matter produced in and transported into the lake to be prevented from decomposing, resulting in higher OC values during cold spells.

## Conclusions

The data presented here demonstrate that temperature changes may have a significant impact on OC burial efficiency. The temperature decrease and likely drier climate conditions caused shifts in the anoxic boundary towards the surface of the lake and thus prevented OC mineralization in an oxic environment. Compared with many previous studies, this study is unbiased with respect to anthropogenic influences, latitude changes or significant vegetation changes, which might have an effect on OC burial efficiency. Our results demonstrate that climate variability was able to trigger mechanisms inherent to the lake, thereby resulting in oscillations in OC burial efficiency.

The Sr/Ca ratio of bulk sediment reflects the formation of aragonite needles in this special lake setting and is a novel approach that can be utilized for paleoclimate reconstructions. We were able to identify several cooling events observed in the wider Mediterranean area, although the unique high resolution of our data enabled us to also identify a number of short-term cold and dry events throughout the 8.3 to 2.6 cal ka BP period that have mainly not been previously observed. Further high-resolution studies on additional archives would be beneficial for investigating their wider regional character.

## Methods

### Collection and extended description of sediment cores

Sediment cores were recovered from the deepest part of the Veliko jezero (M1-A at -45 m) and the deepest part of the second largest basin in Veliko jezero (M2 at -40 m) using a 3 m long piston corer (60 mm diameter UWITEC piston corer) deployed from a floating platform. The M1-A core had a total length of 459 cm, while the M2 core had a total length of 406 cm. Before being split lengthwise and photographed, the entire cores were subjected to magnetic susceptibility loop sensor measurements at 2 cm intervals with a Bartington MS2 magnetic susceptibility system. The split cores were logged for their lithology (smear slides), grain size, textures, structures, clast size and color using both the Munsell color chart and diffuse reflectance measurements (CIELAB–International Commission on Illumination L*a*b*) at 1 cm intervals using an X-rite DTP22 digital swatchbook spectrophotometer. Additionally, the magnetic susceptibility values of the split cores were determined using a Bartington MS2E system at 1 cm intervals. Working halves were subsampled at 1 cm intervals and stored at + 4 °C in plastic bags until further analysis, while the archived halves were stored in D-tubes in a cold chamber at the same temperature. For this study, we used intervals from 114 to 240 cm in core M1-A and from 127 to 266 cm in core M2.

### High-resolution XRF scanning

Both cores M1-A and M2 were analyzed at XRF Core Scanner III (AVAATECH Serial No. 12) at the MARUM Center for Marine Environmental Sciences at the University of Bremen, Germany. Core M1-A was scanned at resolutions of 1 and 0.2 cm, while for Core M2, data were collected every 1 cm. In both cases, generator settings of 10 and 30 kV were applied. A current of 1 mA was used at 30 kV, and 0.2 mA and 0.5 mA at 10 kV were used at 1 cm and 0.2 cm intervals, respectively. The sampling times were 20 s and 30 s for the 1 cm and 0.2 cm intervals, respectively. Before scanning, the split core surface was covered with a 4 µm thin SPEXCerti Prep Ultralene foil to avoid contamination of the XRF measurement unit as well as desiccation of the sediment. The data reported here were acquired by a Canberra X-PIPS Silicon Drift Detector (SDD; Model SXD 15C-150–500) with a 150 eV X-ray resolution, a Canberra Digital Spectrum Analyzer DAS 1000 and an Oxford Instruments 100 W Neptune X-ray tube with rhodium (Rh) target material. Raw data spectra were processed by the analyzing the X-ray spectra by the Iterative Least square software (WIN AXIL) package from Canberra Eurisys. Reliable data (expressed in counts per second (cps)) were collected for the following elements: Br, Rb, Sr, Zr, Al, Si, S, K, Ca, Ti, Mn, and Fe; however, Mo values were considered unreliable because the concentration was close to or below the instruments’ detection limit, and they were validated with an additional ICP-MS analysis on selected samples (Supplementary file, Figure S1). For each core, the RGB and LAB color parameters were obtained at 68 μm resolution. The analysis was performed within 24 h after the core was opened.

### Semiquantitative X-ray diffraction and scanning electron microscopy

To explore whether nonaragonite carbonate phases are present in the studied sediment samples, we performed XRD analyses on samples from Core M1-A. To cover the complete record from this core, we analyzed the mineral composition of 42 composite samples that were defined by homogenization of 3 cm long intervals. The analyses were performed on a PANalytical X’Pert Powder X-ray diffractometer equipped with Ni-filter CuKα radiation, a vertical goniometer with θ/θ geometry and a PIXcel detector. The scan conditions were as follows: 45 kV and 40 mA, ¼ divergence slit and anti-scatter slits, 0.02° 2θ step size, and 2 s time per step. After the samples were ground with mortar and pestle, they were sieved through a 0.4 mm sieve. To reduce the grain size of the material to < 5 μm, powdered samples were ground in McCrone micronizing mills. XRD digital scans were analyzed using the Philips X'Pert High Score search and match function to identify peaks and determine qualitative mineral compositions. Additionally, black and white laminas were examined throughout the cores with scanning electron microscopy (SEM) to study the morphology of aragonite minerals to test their inorganic origin. In the interval from 201 to 214 cm, SEM information was used to infer the morphology and potential nonbiogenic origin of Mg-calcite, which was proven by both XRD and SEM–EDS (energy-dispersive X-ray spectroscopy).

### Elemental analysis of organic matter and stable carbon isotopes

The total organic carbon (TOC) and total nitrogen (TN) of the sediment samples were analyzed using a Thermo Scientific FLASH 2000 Series Nitrogen and Carbon analyzer of the Croatian Geological Survey at a 1 cm resolution in the studied interval. This method allows for direct measurements of the total carbon (TC) and TN. The addition of HCl removes the carbonate component and allows for the determination of TOC. Carbonate was removed by treating 1 g of sediment sample with 12 ml 4 M HCl and heating in a centrifuge tube sitting in a hot block for 2 h. The insoluble residue was washed with Milli-Q water and centrifuged (2x), freeze-dried and weighed. The carbon content of the insoluble residue after HCl treatment is the TOC. The difference between TC and TOC was used for the calculation of TIC, whereas the calcium carbonate (CaCO_3_) content was calculated from the obtained TIC values. The C/N mass ratio was calculated by dividing the TOC and TN. XRD analyses of insoluble residue were used to confirm that carbonates were not present in the samples after HCl treatment. A split of the acid-washed sample was weighed into tin capsules optimized for stable carbon and nitrogen isotope ratio measurements in the Sample Weight Calculator of the Stable Isotope Facility (SIF) at the University of California, USA. The samples were shipped to SIF in sealed evaporation plates with 96 wells. The sediments at SIF were analyzed for δ ^13^C and δ ^15^ N using an Elementar Vario EL Cube (Elementar Analysensysteme GmbH, Hanau, Germany) interfaced with an Isoprime VisION IRMS (Elementar UK Ltd, Cheadle, UK). The isotope data are expressed relative to international standards VPDB (Vienna Pee Dee Belemnite). The long-term standard deviation reported by the SIF is 0.2‰ for δ ^13^C and δ 0.3‰ for δ^15^N. Stable carbon isotopes were measured at a 10 cm resolution.

### Statistical methods

Geochemical data obtained with XRF are compositional data, i.e., all components are parts of the same whole^67^. It is very hard to measure all elements; therefore, in reality, we analyzed a subcomposition, i.e., only some of all possible parts. Prior to the statistical analyses, one should represent data that are originally given as elements of a simplex space in log ratio coordinates^15^. Orthonormal log ratio coordinates (olr) were used^52,68^ to construct geochemical proxies^53^. The orthonormal basis for the olr compositional biplots was constructed using the CoDa pack^69^. The construction of the sample basis was conducted by performing sequential binary partitioning (SBP) of a compositional vector^70^. Prior to the proxy construction, the dimensionality of the problem was reduced with the aid of a compositional biplot^71^, which helped in discarding redundant elements, i.e., those that carry geochemically similar information (have small variance between clr transformed variables). Proxies constructed via this method are free of compositional data restrictions regarding the multivariate statistics and correlation analyses. It is important to stress that the high Mg-calcite interval (group High D) was removed from the correlation analysis of constructed proxies reported in the main paper because of inherently different geochemical affiliations. The rationale for balance construction and compositional biplot interpretation is presented in the Supplementary information.

## Supplementary Information

Supplementary Information.

## Data Availability

The datasets generated during and/or analyzed during the current study are available in the PANGAEA repository, https://doi.org/10.1594/PANGAEA.924331

## References

[1] Mendonça R (2017). Organic carbon burial in global lakes and reservoirs. Nat. Commun..

[2] Gudasz C (2010). Temperature-controlled organic carbon mineralization in lake sediments. Nature.

[3] Larsen S, Andersen T, Hessen DO (2011). Climate change predicted to cause severe increase of organic carbon in lakes. Glob. Chang. Biol..

[4] Anderson NJ, Dietz RD, Engstrom DR (2013). Land-use change, not climate, controls organic carbon burial in lakes. Proc. R. Soc. B Biol. Sci..

[5] Sobek S, Anderson NJ, Bernasconi SM, Del Sontro T (2014). Low organic carbon burial efficiency in arctic lake sediments. J. Geophys. Res. Biogeosci..

[6] Heathcote AJ, Anderson NJ, Prairie YT, Engstrom DR, Del Giorgio PA (2015). Large increases in carbon burial in northern lakes during the Anthropocene. Nat. Commun..

[7] Algesten G (2003). Role of lakes for organic carbon cycling in the boreal zone - Algesten - 2003 - Global Change Biology - Wiley Online Library. Glob. Chang. Biol..

[8] Sobek S, Algesten G, Bergström AK, Jansson M, Tranvik LJ (2003). The catchment and climate regulation of pCO2 in boreal lakes. Glob. Chang. Biol..

[9] Sobek S (2009). Organic carbon burial efficiency in lake sediments controlled by oxygen exposure time and sediment source. Limnol. Oceanogr..

[10] Xu H, Lan J, Liu B, Sheng E, Yeager KM (2013). Modern carbon burial in Lake Qinghai, China.. Appl. Geochem..

[11] Gudasz C, Sobek S, Bastviken D, Koehler B, Tranvik LJ (2015). Temperature sensitivity of organic carbon mineralization in contrasting lake sediments. J. Geophys. Res. G Biogeosciences.

[12] Anderson NJ (2020). Anthropogenic alteration of nutrient supply increases the global freshwater carbon sink. Sci. Adv..

[13] Carey CC, Doubek JP, McClure RP, Hanson PC (2018). Oxygen dynamics control the burial of organic carbon in a eutrophic reservoir. Limnol. Oceanogr. Lett..

[14] Bartosiewicz M (2019). Hot tops, cold bottoms: Synergistic climate warming and shielding effects increase carbon burial in lakes. Limnol. Oceanogr. Lett..

[15] Pawlowsky-Glahn, V., Egozcue, J. J. & Tolosana-Delgado, R. *Modeling and analysis of compositional data*. (Wiley, Chichester, 2015). 10.1017/CBO9781107415324.004

[16] Beck JW (1992). Sea-surface temperature from coral skeletal strontium/calcium ratios. Science.

[17] Dietzel M, Gussone N, Eisenhauer A (2004). Co-precipitation of Sr2+ and Ba2+ with aragonite by membrane diffusion of CO2 between 10 and 50 °C. Chem. Geol..

[18] Kinsman JJ, Holland HD (1969). The co-precipitation of cations with CaCO3 - IV. The co-precipitation of Sr2+ with aragonite between 16" and 96°C. Geochim. Cosmochim. Acta.

[19] Corrège T (2006). Sea surface temperature and salinity reconstruction from coral geochemical tracers. Palaeogeogr. Palaeoclimatol. Palaeoecol..

[20] Villiers SD, Nelson BK, Chivas AR (1994). Biological controls on coral Sr/Ca and δ180 reconstructions of sea surface temperatures. Science.

[21] Cohen AL, Gaetani GA, Lundälv T, Corliss BH, George RY (2006). Compositional variability in a cold-water scleractinian, Lophelia pertusa: New insights into ‘vital effects’. Geochem. Geophys. Geosyst..

[22] de Villiers S, Shen GT, Nelson BK (1994). The Sr Ca-temperature relationship in coralline aragonite: Influence of variability in ( Sr Ca)seawater and skeletal growth parameters. Geochim. Cosmochim. Acta.

[23] Villiers SD (1999). Seawater strontium and Sr=Ca variability in the Atlantic and Pacific oceans. Earth Planet. Sci. Lett..

[24] Cohen AL, Owens KE, Layne GD, Shimizu N (2002). The effect of algal symbionts on the accuracy of Sr/Ca paleotemperatures from coral. Science.

[25] Razum I (2020). Holocene tephra record of Lake Veliko jezero, Croatia : implications for the central Mediterranean tephrostratigraphy and sea level rise. Boreas.

[26] Cacho I, Grimal J, Canals M, Sbaffi L, Shackleton N, Schönfeld J, Z. R. (2001). Variability of the western Mediterranean Sea surface temperature during the last 25000 years and its connection with the Northern Hemisphere climate changes. Paleoceanography.

[27] Sangiorgi F (2003). Holocene seasonal sea-surface temperature variations in the southern Adriatic Sea inferred from a multiproxy approach. J. Quat. Sci..

[28] Combourieu-Nebout N (2013). Holocene vegetation and climate changes in the central Mediterranean inferred from a high-resolution marine pollen record (Adriatic Sea). Clim. Past.

[29] Siani G, Magny M, Paterne M, Debret M, Fontugne M (2013). Paleohydrology reconstruction and Holocene climate variability in the South Adriatic Sea. Clim. Past.

[30] Regattieri E (2014). Lateglacial to Holocene trace element record (Ba, Mg, Sr) from Corchia Cave (Apuan Alps, central Italy): Paleoenvironmental implications. J. Quat. Sci..

[31] Samartin S (2017). Warm Mediterranean mid-Holocene summers inferred from fossil midge assemblages. Nat. Geosci..

[32] Vlahović I, Tišljar J, Velić I, Matičec D (2005). Evolution of the Adriatic Carbonate Platform: Palaeogeography, main events and depositional dynamics. Palaeogeogr. Palaeoclimatol. Palaeoecol..

[33] Wunsam S, Schmidt R, Müller J (1999). Holocene lake development of two dalmatian lagoons (Malo and Veliko Jezero, Isle of Mljet) in respect to changes in Adriatic sea level and climate. Palaeogeogr. Palaeoclimatol. Palaeoecol..

[34] Blaauw, M. & Christen, A. rbacon: Age-Depth Modelling using Bayesian Statistics. *R Packag. version 2.3.4.* (2018).

[35] Morse JW, Wang Q, Tsio MY (1997). Influences of temperature and Mg: Ca ratio on CaCO3 precipitates from seawater. Geology.

[36] Bischoff JL, Fyfe WS (1968). Catalysis, inhibition, and the calcite-aragonite problem; [Part] 1, The aragonite-calcite transformation.. Am. J. Sci..

[37] Berner RA (1975). The role of magnesium in the crystal growth of calcite and aragonite from sea water. Geochim. Cosmochim. Acta.

[38] Gaetani GA, Cohen AL (2006). Element partitioning during precipitation of aragonite from seawater: A framework for understanding paleoproxies. Geochim. Cosmochim. Acta.

[39] Mucci A, Canuel R, Zhong S (1989). The solubility of calcite and aragonite in sulfate- free seawater and the seeded growth kinetics and composition of the precipitates at 25 °C. Chem. Geol..

[40] Zhong S, Mucci A (1989). Calcite and aragonite precipitation from seawater solutions of various salinities: precipitation rates and overgrowth compositions. Chem. Geol..

[41] Sondi I, Juračić M (2010). Whiting events and the formation of aragonite in Mediterranean karstic marine lakes: New evidence on its biologically induced inorganic origin. Sedimentology.

[42] Bar-Matthews M, Ayalon A, Kaufman A (1997). Late Quaternary Paleoclimate in the Eastern Mediterranean Region from Stable Isotope Analysis of Speleothems at Soreq Cave, Israel.. Quat. Res..

[43] Kallel N (1997). Enhanced rainfall in the Mediterranean region during the last Sapropel Event. Oceanol. Acta.

[44] Bar-Matthews, M., Ayalon, A. & Kaufman, A. Middle to Late Holocene (6,500 Yr. Period) Paleoclimate in the Eastern Mediterranean Region from Stable Isotopic Composition of Speleothems from Soreq Cave, Israel. 203–214 (1998). 10.1007/978-94-017-3659-6_9

[45] Bar-Matthews M, Ayalon A (2011). Mid-holocene climate variations revealed by high-resolution speleothem records from soreq cave, israel and their correlation with cultural changes. Holocene.

[46] Magny M (2012). Contrasting patterns of precipitation seasonality during the Holocene in the south- and north-central Mediterranean. J. Quat. Sci..

[47] Zhornyak LV (2011). Stratigraphic evidence for a ‘ pluvial phase’ between ca 8200–7100 ka from Renella cave (Central Italy). Quat. Sci. Rev..

[48] Kitano Y, Kanamori N, Oomori T (1971). Measurements of distribution coefficients of strontium and barium between carbonate precipitate and solution —Abnormally high values of distribution coefficients measured at early stages of carbonate formation. Geochem. J..

[49] Martin J, Meybeck M (1979). Elemental mass-balance of material carried by major world rivers. Mar. Chem..

[50] Fletcher WJ, Debret M, Sanchez Goñi M (2012). Mid-Holocene emergence of a lowfrequency millennial oscillation in western Mediterranean climate: Implications for past dynamics of the North Atlantic atmospheric westerlies. The Holocene.

[51] Bond G (2001). Persistent solar influence on north atlantic climate during the Holocene. Science.

[52] Egozcue JJ, Pawlowsky-Glahn V (2019). Compositional data: the sample space and its structure. TEST.

[53] Razum I, Miko S, Ilijanić N, Hasan O, Šparica Miko M, Brunović D, Pawlowsky-Glahn V (2020). A compositional approach to the reconstruction of geochemical processes involved in the evolution of Holocene marine flooded coastal karst basins (Mljet Island, Croatia). Appl. Geochemistry.

[54] Arz HW, Lamy F, Pätzold J (2006). A pronounced dry event recorded around 4.2 ka in brine sediments from the northern Red Sea. Quat. Res..

[55] Drysdale R (2006). Late Holocene drought responsible for the collapse of Old World civilizations is recorded in an Italian cave flowstone. Geology.

[56] Magny M (2009). Possible complexity of the climatic event around 4300–3800 cal: BP in the central and western Mediterranean. Holocene.

[57] Bini M (2019). The 42 ka BP Event in the Mediterranean region: An overview. Clim. Past.

[58] Blenckner T (2005). A conceptual model of climate-related effects on lake ecosystems. Hydrobiologia.

[59] Meyers PA, Ishiwatari R (1993). Lacustrine organic geochemistry-an overview of indicators of organic matter sources and diagenesis in lake sediments. Org. Geochem..

[60] Meyers PA (1994). Preservation of elemental and isotopic source identification of sedimentary organic matter. Chem. Geol..

[61] Lamb AL, Wilson GP, Leng MJ (2006). A review of coastal palaeoclimate and relative sea-level reconstructions using δ13C and C/N ratios in organic material. Earth-Science Rev..

[62] Jahns S, Bogaard C (1998). New palynological and tephrostratigraphical investigations of two salt lagoons on the island of Mljet, south Dalmatia, Croatia.. Veg. Hist. Archaeobot..

[63] Sampei Y, Matsumoto E (2001). C / N ratios in a sediment core from Nakaumi Lagoon, southwest Japan — usefulness as an organic source indicator —. Geochem. J..

[64] Zadereev ES, Tolomeev AP, Drobotov AV, Kolmakova AA (2014). Impact of weather variability on spatial and seasonal dynamics of dissolved and suspended nutrients in water column of meromictic Lake Shira. Contemp. Probl. Ecol..

[65] Cantin A, Beisner BE, Gunn JM, Prairie YT, Winter JG (2011). Effects of thermocline deepening on lake plankton communities. Can. J. Fish. Aquat. Sci..

[66] Benović A (2000). Ecological characteristics of the Mljet Island seawater lakes ( South Adriatic Sea ) with special reference to their resident populations of medusae. Sci. Mar..

[67] Aitchison J (1982). The Statistical Analysis of Compositional Data. J. R. Stat. Soc. Ser. B.

[68] Egozcue JJ, Pawlowsky Glahn V, Mateu-Figueras G, Barceló Vidal C (2003). Isometric logratio for compositional data analysis. Math. Geol..

[69] Comas, M. & Thió-Henestrosa, S. CoDaPack 2.0: a stand-alone, multi-platform compositional software. in *4th International Workshop on Compositional Data Analysis* (eds. Egozcue, J. J., Tolosana-Delgado, R. & Ortego, M. I.) 1–10 (2011).

[70] Egozcue, J. & Pawlowsky-Glahn, V. CoDa-dendrogram: A new exploratory tool. *Compos. Data Anal. Work. - CoDaWork’05, Proc.* 1–10 (2005).

[71] Aitchison J, Greenacre M (2002). Biplots of compositional data. *J. R*. Stat. Soc. Ser. C Appl. Stat..

[72] Melbourne, P. A thermochemical study of vaterite. **37**, (1973).

