# Supplementary material for: Past climate variations recorded in needle-like aragonites correlate with organic carbon burial efficiency as revealed by lake sediments in Croatia
Source: Sci Rep. 2021 Apr 7;11:7568. doi: 10.1038/s41598-021-87166-2 (PMC8027459; doi:10.1038/s41598-021-87166-2)
Supplement: Supplementary file 1 — Supplementary Information. [file 41598_2021_87166_MOESM1_ESM.pdf]

Supporting Information for

Past climate variations recorded in needle-like aragonites correlate with organic carbon burial efficiency as revealed by lake sediments in Croatia

Ivan Razum[1], Petra Bajo[2], Dea Brunović[2], Nikolina Ilijanić[2], Ozren Hasan[2], Ursula Röhl[3], Martina Šparica Miko[2] and Slobodan Miko[2]

[1]Croatian Natural History Museum, Demetrova 1, 10000 Zagreb, Croatia, [2]Croatian Geological Survey, Sachsova 2, 10000 Zagreb, Croatia, [3]MARUM-Center for Marine Environmental Sciences, University of Bremen, Leobener Strasse, 28359 Bremen, Germany

Contents of this file

Text S1
Figures S1 to S4
Tables S1 to S2

Additional Supporting Information (datasets stored in Pangaea repository)

PANGAEA, https://doi.org/10.1594/PANGAEA.924331

Captions for Datasets S1 to S6

Introduction

In this supporting information we provide comparison of a Mo trend in the studied interval derived from µ-XRF and ICP-MS method (Figure S1). Statistical treatment of the data and rationale for the construction of proxies (Text S1, Figures S2-S3, Table S1) and correlation matrix between modelled proxies (Table S2). Additionally, age-depth model of the core M2 (Figure S4) is presented. Datasets containing results of µ-XRF, organic carbon, XRD and stable isotopes are stored in Pangaea repository.

Text S1 (balance construction)

2.1. Rationale for balance construction

'Balance 1' is the log ratio of elements affiliated with carbonates against all other elements and it can be interpreted as proxy for relative proportion of carbonates i.e. high balance values indicate where detrital component is least represented.

'Balance 2' is the log ratio of Sr over Ca, which is used as proxy for relative changes in paleotemperature since Sr content is mainly temperature dependent. The Sr/Ca ratio of aragonite is typically used as a proxy for SST variability [1] because incorporation of Sr into aragonite corral skeletons as well as in inorganic aragonite is temperature dependent, i.e. an increase in temperature lowers the Sr/Ca ratio in inorganic aragonite [2,3] and in coral skeletons [4].

'Balance 3' is the log ratio of Mo over typically detrital elements (Fe, Zr, Al). It is interpreted as a proxy for paleoredox conditions. High balance values indicate relative Mo enrichment compared to detrital components. There are two options for Mo enrichment in sediments: Either by adsorption at pre-mobilized Mn-oxide in an oxygenated environment [5–7], not being an option for our studied sediments, which were under constant anoxic conditions [8]. Alternatively, and in the presence of sulphides Mo may has been fixed in the sediment via the formation of thiomolybdates, that were in the following bonded to Fe particles, sulphur-rich organic matter or iron sulphides [9,10]. Mo reduction or deposition may occur in a water column under anoxic conditions. Organic matter plays an important role because if it is rich in S, it will easily bind with Mo and fixate it to the sediment [11], which links the cycle of organic matter and the concentration of Mo [12].

'Balance 4' is the log ratio of Fe over Zr and Al. Since Fe concentrations may also be redox conditioned [11] this balance tells us were Fe was enriched compared to Zr and Al, it is most likely that Fe enrichment is a consequence of sulphide deposition under anoxic conditions. Relatively high correlation ($r = 0.7$) with 'balance 3' proves the proposition that these two independent proxies both reflect paleoredox conditions.

'Balance 5' is the log ratio of Zr over Al. This proxy is interpreted as an indicator for aeolian activity. Zr is typically more enriched in coarser fraction i.e. sand and silt, which due to the lack on water inflow into the lake are transported in the center of the basin (location of the core) by wind. Thus, relative enrichment of Zr compared to Al (generally more pronounced in the finer fraction) is proxy for enhanced aeolian activity.

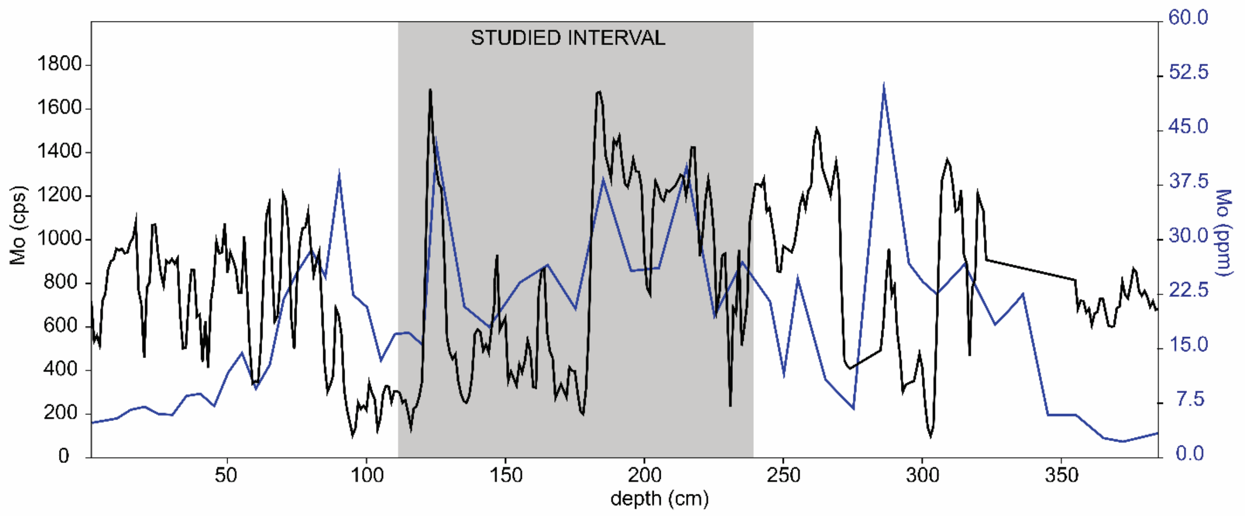

Figure S1. Comparison of Mo trend obtained with ICP-MS (blue line) and XRF (black line) throughout the core. It is visible that in the studied interval trends are perfectly matched, proving the reliability of the XRF data. Data for Mo (ppm) is published before. [8]

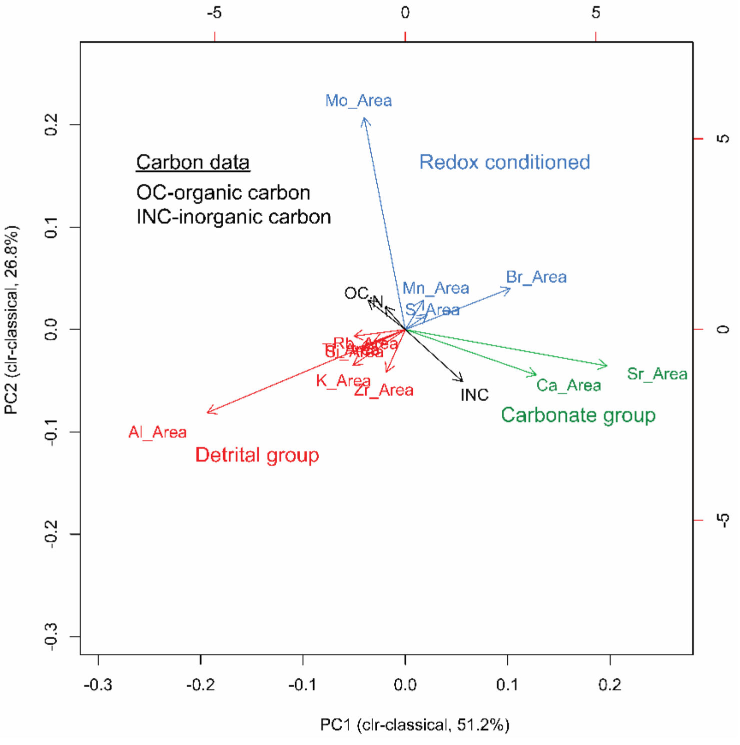

Figure S2. Compositional biplot made in R package robCompositions [13], using the mult_comp argument for two groups of compositional data i.e. XRF (M1-A 1cm resolution) and elemental analysis of organic matter. Three dominant groups of elements are visible and their correlation with carbon (OC, INC) and nitrogen. Biplot was used along with a variation matrix to reduce the number of elements used for proxy construction, i.e. elements with low variation carry geochemically similar information and are redundant.

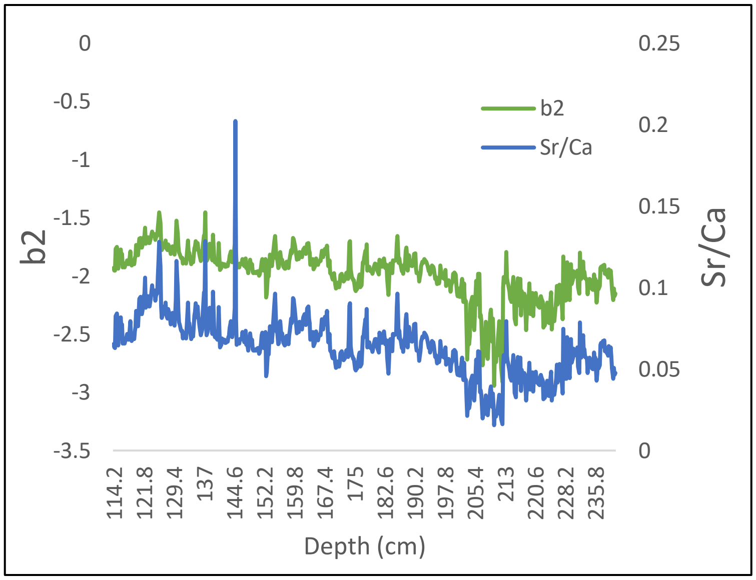

Figure S3. Comparison of b2 and Sr/Ca trend to show that constructed relative paleoclimate proxy needed for correlation analysis reflects the Sr/Ca which is mandatory for the Fig 4 in main text.

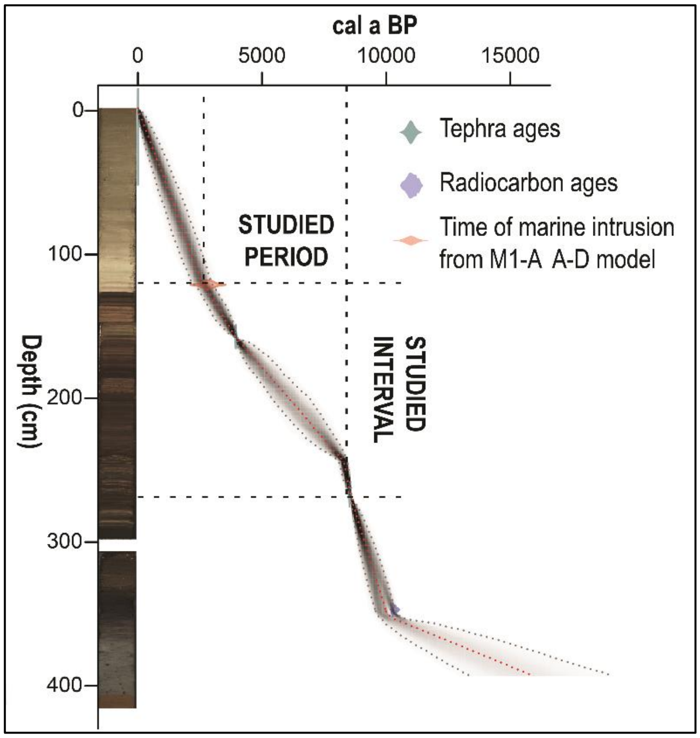

Figure S4. Age-Depth model of M2 core.

Table S1. Binary partition used for OLR(ILR) transformation and construction of proxies.

ILR binary partition:						
**Sr_Area**	**Zr_Area**	**Al_Area**	**Mo_Area**	**Fe_Area**	**Ca_Area**	**Balance Interpretation**
1	-1	-1	-1	-1	1	b1 relative amount of carbonates
1	0	0	0	0	-1	b2 paleoclimate (Sr/Ca)
0	-1	-1	1	-1	0	b3 paleoredox 1
0	-1	-1	0	1	0	b4 paleoredox 2
0	1	-1	0	0	0	b5 aeolian intensity

Table S2. Correlation matrices between proxies (balances) for each different geochemical environment. In the main text reported correlations are from the LOW D (Aragonite) group where Sr/Ca is mainly temperature conditioned

LOW D (Sr/Ca-temperature conditioned) Aragonite					
**Correlation:**					
	**b1**	**b2**	**b3**	**b4**	**b5**
**b1**	1	0.4197	0.4583	0.1774	0.3712
**b2**	0.4197	1	0.5478	0.5154	0.5605
**b3**	0.4583	0.5478	1	0.7013	0.3217
**b4**	0.1774	0.5154	0.7013	1	0.2404
**b5**	0.3712	0.5605	0.3217	0.2404	1
**HIGH D**	**(Sr/Ca-increased precipitation) Mg-Calcite**				
**Correlation:**					
	**b1**	**b2**	**b3**	**b4**	**b5**
**b1**	1	0.0849	0.722	-0.0012	0.2322
**b2**	0.0849	1	0.1172	0.4265	0.1258
**b3**	0.722	0.1172	1	0.3476	0.4077
**b4**	-0.0012	0.4265	0.3476	1	0.2377
**b5**	0.2322	0.1258	0.4077	0.2377	1

Data Set S1. Results of a XRF analysis of the core M1-A in 2 mm resolution (ds01).

Data Set S2. Results of a XRF analysis of the core M1-A in 1 cm resolution (ds02)

Data Set S3. Results of a XRF analysis of the core M2 in 1 cm resolution (ds03)

Data Set S4. Results of a total carbon and nitrogen analysis (ds04)

Data Set S5. Results $\delta^{13}C$ analysis (ds05)

Data Set S6. Results of XRD analysis of the core M1-A (ds06)

References

1. Beck, J. W. *et al.* Sea-Surface Temperature from Coral Skeletal Strontium/Calcium Ratios. *Science (80-.).* **257**, 0–3 (1992).
2. Dietzel, M., Gussone, N. & Eisenhauer, A. Co-precipitation of Sr^{2+} and Ba^{2+} with aragonite by membrane diffusion of CO_2 between 10 and 50 °C. *Chem. Geol.* **203**, 139–151 (2004).
3. Kinsman, J. J. & Holland, H. D. The co-precipitation of cations with CaCO3 - IV. The co-precipitation of Sr^{2+} with aragonite between 16° and 96°C. *Geochim. Cosmochim. Acta* **33**, 1–17 (1969).
4. Corrège, T. Sea surface temperature and salinity reconstruction from coral geochemical tracers. *Palaeogeogr. Palaeoclimatol. Palaeoecol.* **232**, 408–428 (2006).
5. Scott, C. & Lyons, T. W. Contrasting molybdenum cycling and isotopic properties in euxinic versus non-euxinic sediments and sedimentary rocks: Refining the paleoproxies. *Chem. Geol.* **324–325**, 19–27 (2012).
6. Shimmield, G. B. & Price, N. B. The behaviour of molybdenum and manganese during early sediment diagenesis - offshore Baja California, Mexico. *Mar. Chem.* **19**, 261–280 (1986).
7. Zheng, Y., Anderson, R. F., Van Geen, A. & Kuwabara, J. Authigenic molybdenum formation in marine sediments: A link to pore water sulfide in the Santa Barbara Basin. *Geochim. Cosmochim. Acta* **64**, 4165–4178 (2000).
8. Razum, I., Miko, S., Ilijanić, N., Hasan, O., Šparica Miko, M., Brunović, D., Pawlowsky-Glahn, V. A compositional approach to the reconstruction of geochemical processes involved in the evolution of Holocene marine flooded coastal karst basins (Mljet Island, Croatia). *Appl. Geochemistry* **116**, 104574 (2020).
9. Helz, G. R. *et al.* Mechanism of molybdenum removal from the sea and its concentration in black shales: EXAFS evidence. *Geochim. Cosmochim. Acta* **60**, 3631–3642 (1996).
10. Vorlicek, T. P., Kahn, M. D., Kasuya, Y. & Helz, G. R. Capture of molybdenum in pyrite-forming sediments: Role of ligand-induced reduction by polysulfides. *Geochim. Cosmochim. Acta* **68**, 547–556 (2004).
11. Tribovillard, N., Algeo, T. J., Lyons, T. & Riboulleau, A. Trace metals as paleoredox and paleoproductivity proxies: An update. *Chem. Geol.* **232**, 12–32 (2006).
12. McManus, J. *et al.* Molybdenum and uranium geochemistry in continental margin sediments: Paleoproxy potential. *Geochim. Cosmochim. Acta* **70**, 4643–4662 (2006).
13. Templ, M., Hron, K. & Filzmoser, P. Package ‘ robCompositions ’. in *Data Analysis. Theory and Applications* (eds. Pawlowsky-Glahn, V. & Buccianti, A.) 341–345 (John Wiley & Sons, Chichester (UK), 2011).
